# Etymologia

**DOI:** 10.3201/eid1701.ET1702

**Published:** 2011-02

## *Naegleria fowleri *[nə′gliəriə fau(ə)l′∙ər∙ī]

From F.P.O. Nägler, an early 20th century bacteriologist, and Malcolm Fowler, an Australian physician. In 1912, A. Alexeieff proposed a new genus for questionable amoeboid forms, which he named Nägleria in honor of Nägler’s work in identifying amoebae that pass through a biflagellate stage. Fifty-three years later, a report from Australia described human meningoencephalitis caused by an amebo-flagellate, later recognized as a member of *Naegleri*a. In 1970, the pathogen was designated *Naegleria fowleri* after Fowler, who obtained one of the first isolates from human brain tissue.

**Sources:** Alexeieff A. Sur less caractères cytologiques et la systématique des amibes du groupe limax (Naegleria nov gen et Hartmannia nov. gen) et des amibes parasites des vertebras (Proctamoeba nov. gen). Bull de la Soc Zool de France. 1912; 37:55; Calkins GN. Genera and species of ameba. In: Transactions of the Fifteenth International Congress on Hygiene and Demography, Vol. II, Washington, September 23–28, 1912. Washington: General Printing Office; 1913; Fowler M, Carter RF. Acute pyogenic meningitis probably due to Acanthamoeba sp.: a preliminary report. BMJ. 1965;2:740–2. PubMed DOI: 10.1136/bmj.2.5464.734-a; Marciano-Cabral F. Biology of Naegleria spp. Microbiol Rev. 1998;52:114–33. PubMed

